# Real-Time Expression Analysis of Selected *Anticarsia gemmatalis* *multiple nucleopolyhedrovirus* Gene Promoters during Infection of Permissive, Semipermissive and Nonpermissive Cell Lines

**DOI:** 10.3390/v9060132

**Published:** 2017-05-31

**Authors:** Fabricio da Silva Morgado, Daniel Mendes Pereira Ardisson-Araújo, Bergmann Morais Ribeiro

**Affiliations:** 1 Laboratory of Baculovirus, Cell Biology Department, University of Brasília, 70910–900 Brasília-DF, Brazil; fabsmorga@gmail.com; 2 Laboratory of Insect Virology, Department of Biochemistry and Molecular Biology, Federal University of Santa Maria, 97105–900 Santa Maria-RS, Brazil; daniel_ardisson@yahoo.com.br

**Keywords:** Baculovirus, *Anticarsia gemmatalis*, cell infection, permissive, nonpermissive, host range, promoter, hyperexpression, apoptosis

## Abstract

Baculovirus infection follows a transcriptionally controlled sequence of gene expression that occurs by activation of different viral gene promoter sequences during infection. This sequence of promoter activation may be disrupted by cellular defenses against viral infection, which might interfere with viral progeny formation. In this work, the activity of the *ie1*, *gp64*, *lef-1*, *vp39*, *p6.9* and *polh* promoters of the *Anticarsia gemmatalis multiple nucleopolyhedrovirus* was assessed during infection of permissive, semipermissive and nonpermissive cell lines by a novel methodology that detects reporter protein luminescence in real-time. This technique allowed us to characterize in rich detail the AgMNPV promoters in permissive cell lines and revealed differential profiles of expression in cells with limited permissivity that correlate well with limitations in viral DNA replication. Semipermissive and nonpermissive cell lines presented delays and restrictions in late and very late promoter expression. Cells undergoing apoptosis did not inhibit late gene expression; however, viral progeny formation is severely affected. This work demonstrates the application of the real-time luminescence detection methodology and how the promoter expression profile may be used to diagnose cellular permissivity to baculovirus infection.

## 1. Introduction

Baculoviruses are circular double-stranded DNA viruses that infect larvae of lepidopteran, dipteran and hymenopteran insects. These viruses are used as biological control agents of insect pests [1] and expression vectors for recombinant protein production in insects and insect cells [2]. They produce two infectious phenotypes at different times during infection, the extracellular virus (also named as budded virus, BV) and the occlusion-derived virus (ODV). The BV phenotype is produced at late times of infection and is responsible for the spread of the virus within the insect body. The ODV is produced at very late times of infection and is occluded into a protein matrix called the occlusion body or polyhedra (OB) which is responsible for the horizontal transmission of the virus in the field [2,3,4].

The most extensively used virus insecticide in the world was the baculovirus *Anticarsia gemmatalis multiple nucleopolyhedrovirus* (AgMNPV) to control the velvetbean caterpillar *Anticarsia gemmatalis* (Lepidoptera: Noctuidae) [1]. AgMNPV is phylogenetically close to both *Choristoneura fumiferana defective MNPV* (CfDEFMNPV, [5]) and *Condylorrhiza vestigialis MNPV* (CoveMNPV, [6]) but has a more distant relationship to the most studied species of baculovirus, the *Autographa californica MNPV* (AcMNPV) [7,8]. There are many distinct differences between the AgMNPV genome and other baculoviruses which makes it an interesting object of study. It naturally lacks the viral protease cathepsin (V-CATH) and the hydrolase chitinase (CHI-A, [9]). This feature also makes it an interesting alternative as a protein expression vector since the *v-cath* gene product has been shown to compromise protein production when using the AcMNPV as an expression vector [10,11].

Insect cell lines have varied susceptibilities to baculovirus infection in vitro, ranging from permissive (i.e., large amounts of matured viral phenotypes are produced) to nonpermissive (i.e., blockage of virus replication and mature virus formation). Cell lines derived from the velvetbean caterpillar, *A. gemmatalis* (UFL-Ag-286 or UFLAg) and the cabbage looper, *Trichoplusia ni* (BTI-Tn-5B1-4 or Tn5B) are permissive to AgMNPV infection allowing for high titers of BVs and OB production [12,13,14,15]. A cell line derived from the fall armyworm (Sf9 from *Spodoptera frugiperda*) was found previously to be semipermissive due to a reduced production of polyhedra [14] and 100-fold lower BV titer as compared to UFLAg [16]. The infectivity of AgMNPV to IPLB-Ld652Y (Ld652Y) derived from the gipsy moth (*Lymantria dispar*) has been defined as nonpermissive due to reduced viral replication, reduced infective progeny production and lack of OB formation [14]. In the silkworm *Bombyx mori* derived cell line (Bm5), neither viral replication nor viral progeny were detected, making it nonpermissive to AgMNPV [13,14]. No data exists on the permissivity status of this virus during infection of the tomato looper, *Chrysodeixis chalcites* cell line WU-Cce-1 (Chch).

Gene expression during viral infection follows a transcriptionally controlled and sequential pattern that begins at the immediate early phase. This phase of the infection focuses on establishing control over the cellular apparatus, hijacking the cell’s own transcription machinery to transactivate viral gene transcription [17] and disarming cellular defenses [18,19]. This is followed by the delayed early phase, marked by the production of the viral replication [20] and viral RNA transcription machinery [21,22]. Delayed early genes are transcribed by a combination of the host RNA pol II complex with viral transactivator proteins [23,24]. The host RNA pol II protein complex binds to AT rich sequences in the early gene promoters that possess the nucleotide sequence CAGT, a common early transcription start site (TSS) motif [25], but it is not considered essential for transcription of early genes [26]. Another common motif found in early promoters is the sequence TATAA (TATA box) at average −32 base pair (bp) upstream of the early TSS [26], that has been shown to control start site selection and efficiency [24,27]. The combination of a TATA box and CAGT motifs makes up what is considered to be a “canonical early promoter”. By definition, the early phase of infection ends as viral DNA replication begins. In order to produce large amounts of viral progeny, viral DNA replication and the viral RNA polymerase act in concert to promote hyperexpression of late and very-late genes [2]. Late genes generally express structural proteins such as the capsid protein VP39 and the viral DNA binding protein P6.9, two of the main components of the matured virions [28]. This is only possible due to the specific and high transcription rate of the viral RNA polymerase [29,30,31] over the late and very late gene promoters [32,33], having as a common TSS the TAAG sequence motif [26,29].

In order to better understand the baculovirus transcription scheme of AgMNPV during infection of insect cells lines with different susceptibilities and characterize the expression of selected promoters, recombinant AgMNPVs were constructed containing the *ie1*, *gp64*, *vp39*, *lef1*, *p6.9* and *polyhedrin* promoters controlling the expression of the firefly *luciferase* gene (*fluc*). Reporter protein luminescence was measured in real-time during cell infection using a novel methodology. The resulting data demonstrates that the dynamics of gene expression during infection proceeds in a conserved sequence of promoter activation in all cell lines, but the defense mechanisms against infection within the host cells have a disruptive effect on the timing and intensity of expression.

## 2. Materials and Methods

### 2.1. Insect Cells and Viruses

The in vitro grown insect cell lines UFL-Ag-286 (UFLAg) derived of *Anticarsia gemmatalis* [34], BTI-Tn-5B1-4 (Tn5B, [35]), Sf9 a *Spodoptera frugiperda* IPLB-Sf21-AE clonal isolate [36] and WU-Cce-1 (Chch, [37]) were grown in TC-100 medium (Vitrocell, Campinas, São Paulo, Brazil) supplemented with 10% FBS (Invitrogen, Carlsbad, CA, USA) . IPLB-Ld652Y (Ld652Y, [38]) and Bm5 [39] were grown in Grace’s medium supplemented with 10% FBS. The baculovirus AgMNPV isolate 2D [40], and the recombinant baculovirus vAgGAL, derived from AgMNPV-2D which has the *β-gal* gene replacing the *polyhedrin* gene [41], were also used in this work.

### 2.2. Recombinant Baculovirus Construction

The *Photinus pyralis* firefly *luciferase* gene (*fluc*) was isolated from the plasmid pGEM-luc (Promega, Madison, WI, USA) by Polymerase Chain Reaction (PCR) using specific oligonucleotides FLUCFWR and FLUCREV (Table 1), following the recommendations of the Taq DNA Polymerase manufacturer (Promega). The reverse oligonucleotide (FLUCREV) added a polyA tail signal (AATAAA) to the amplified DNA fragment. This was cloned into pGEM-T Easy vector (Promega) following the manufacturer’s instructions, generating the plasmid pGEMFLUC [42].

All AgMNPV promoters were amplified by PCR using purified viral DNA as template [4] and specific oligonucleotides (Table 1). The main criteria used to select the promoter sequences was to isolate as much of the intergenic regions upstream of the open reading frame (ORF)`s initiation codon as possible, taking into consideration the presence of restriction sites that might interfere into the cloning scheme. The AgMNPV immediate early 1 (*ie1*) gene promoter was cloned into the pGEM-T Easy plasmid and then subcloned by *Eco*RI restriction enzyme digestion and ligation into pBluescript [19]. The *fluc* gene was removed from pGEMFLUC by *Bam*HI and subcloned downstream of the *ie1* promoter in pBluescript plasmid, generating the plasmid pBSIE1FLUC. The *gp64* and *lef-1* gene promoters were also subcloned into the pGEMFLUC plasmid, generating the plasmids pLEF1FLUC and pGP64FLUC.

The constructs containing the promoters fused with the *fluc* gene (pIE1FLUC, pLEF1FLUC and pGP64FLUC) were subcloned by restriction enzyme digestion with the enzymes *Apa*I and *Sac*I, followed by T4 DNA polymerase blunting and DNA ligation into the homologous recombination vector p2100, which contains a unique *Eco*RV site 158 bp upstream of the polyhedrin (*polh*) ORF start codon [43]. The resulting plasmids, p2100prGP64FLUC, p2100prLEF1FLUC and p2100prIE1FLUC, contain the promoter-*fluc* constructs and a complete *polh* gene within a 2100 bp fragment of the *polh* gene locus of AgMNPV.

The promoters of the *vp39* and *p6.9* genes were also isolated by PCR (Table 1). To the sense primers, it was added a *Hind*III restriction site and in the antisense primers a *Xma*I restriction site. The PCR amplicons were digested and ligated into the p2100prIE1FLUC plasmid that was double digested by *Hind*III and *Xma*I enzymes, replacing the IE1 promoter. This generated the transfer vectors: p2100prVP39FLUC and p2100prP69FLUC. The transfer vector containing the *polh* promoter (p2100prPOLHFLUC) was constructed using the same strategy, as described in previous work [42].

The DNA of the recombinant baculovirus vAgGAL was obtained by infecting UFLAg cells, collecting the supernatant after 6 days post infection (d p.i.) and centrifuging at 1000× *g* for 5 min to remove cells and debris. This viral inoculum was subjected to sucrose cushion centrifugation as described elsewhere [4], to concentrate viral particles. The pellet of this centrifugation was resuspended in viral disruption buffer containing Proteinase K (500 µg/mL) and the mixture was incubated for 4 h at 37 °C. This material was subjected to phenol-chloroform extraction of nucleic acids [4]. The viral DNA was resuspended in sterile water and quantified by agarose gel (0.8%) electrophoresis [44].

**Figure 1:**
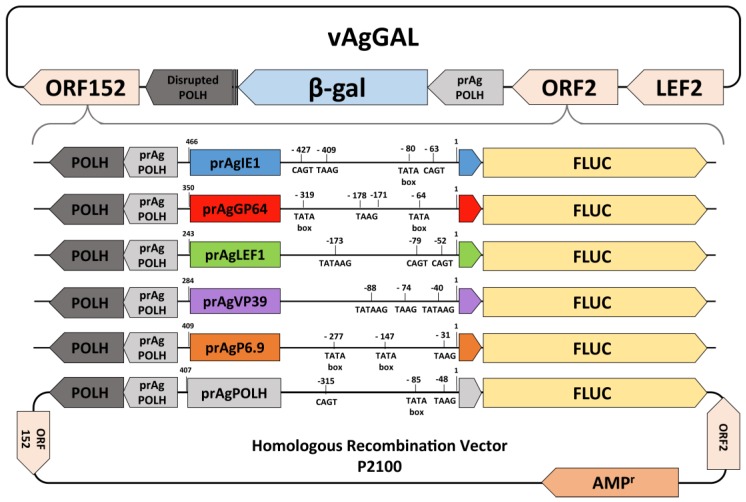
Schematic representation of the different recombinant viruses constructed and used in the experiments. The vAgGAL genome contains an insertion of the *β-Galactosidase* gene (*β-Gal*) within the *polh* locus, disrupting the ORF of the *polh* gene. All promoters were isolated from the AgMNPV-2D genome by polymerase chain reaction (PCR) and subcloned upstream of the *fluc* gene in the transfer vector p2100, that contains a 2100 bp fragment of the AgMNPV polyhedrin locus, with a full length *polh* gene and promoter. Early and late sequence motifs are presented for each promoter. The upstream position in bp of each motif in relation to the ORF start site is also displayed.

To generate recombinant AgMNPV baculoviruses, liposome mediated cotransfections of the vAgGAL viral DNA and each of the transfer vectors into UFLAg cells were carried out (Figure 1). The cotransfections were made using Cellfectin II according to the manufacturer’s instructions (Invitrogen). In brief, for each transfection, 1 µg of viral DNA and 1 µg of transfer vector DNA and 8 µL of Cellfectin II reagent (Invitrogen) were mixed in 100 µL of TC100 serum-free medium and incubated for 10 min at room temperature. The liposome-DNA mixture was added to a 35 mm plate containing 2 × 10^5^/mL UFLAg cells. The resulting recombinant viruses were isolated by end-point dilution and identified by light microscopy for the presence of cells containing polyhedra [4]. After at least four rounds of sequential end-point dilutions, the viral inoculum was amplified in UFLAg cells. Recombinant viruses were titered using the tissue culture infectious dose (TCID_50_) method [45].

### 2.3. Real-Time Luciferase Assay

One day before the assay, the insect cell growing medium is aspirated and a new batch is added, supplemented with 20 mM D-Luciferin (Sigma-Aldrich, St. Louis, MO, USA). At the beginning of the assay, the cells are quantified by microscopic evaluation and diluted to reach 2 × 10^5^ cells/mL, then added to sterile 96 well Costar opaque bottom black plates (used to prevent luminescence leakage between wells). The titered virus inoculae are added to each well, diluted to reach a multiplicity of infection (MOI) 10 at 10 µL doses per well, in triplicates for each virus and cell type. The plate is placed in a GloMax 96 Luminometer (Promega) and the machine is set up to take readings at 999 s intervals and 5 s of integration time at room temperature (25 °C). Results are presented in Relative Light Units (RLU) and 20 RLU is considered the baseline value for a signal above background levels.

### 2.4. Viral DNA Quantification by Quantitative Polymerase Chain Reaction

Absolute quantification of viral DNA by quantitative polymerase chain reaction (qPCR) was used to estimate intracellular viral replication and extracellular budded virus concentration. This assay was conducted by infecting 4 × 10^4^ cells at MOI 10 in triplicates for each cell-line virus combination. After 1 h incubation, excess virus was removed by collecting the viral inoculum, washing the cells and replacing with fresh medium which constitutes the start of infection time. At the time of collection (1, 3, 6, 9, 12, 24 and 48 h post infection: hpi) the supernatants were collected and preserved at 4 °C while the adherent cells were washed and resuspended with 100 µL of PBS (pH 7).

To isolate intracellular DNA, the collected cells were pelleted by centrifugation (1000× *g*/10 min) and resuspended with TNES buffer (10 mM Tris pH 7.5, 400 mM NaCl, 100 mM EDTA and 0.6% SDS), and 5 µL of Proteinase K (10 mg/mL) was added. This was incubated at 55 °C for 4 h. After incubation, 30 µL of NaCl 5 M was added and the solution was centrifuged (12,000 × *g* for 10 min at 4 °C) to pellet the precipitate. The supernatant was collected and 200 µL of absolute ethanol was added. This solution was centrifuged (12,000 × *g* for 15 min at 4 °C) to pellet total DNA. The pellet was washed with 70% ethanol and then resuspended in 20 µL of sterile water.

To isolate extracellular viral DNA contained in BVs we modified the method used by Mukawa & Goto [46]. Thirty µL of the collected insect cell medium was centrifuged to collect cellular debris (1000× *g* for 10 min). To the supernatant was added 125 µL of TE (pH 8), 125 µL of SDS 2% and 5 µL of Proteinase K (10 mg/mL); this was incubated at room temperature for 16 h. After incubation, 100 µL of ammonium acetate (4 M) was added and the solution was mixed and centrifuged to pellet precipitants (12,000× *g* for 10 min at 4 °C). To the supernatant was added 2 volumes of cold absolute ethanol and this was then centrifuged to pellet viral DNA (12,000× *g*/10 min). The DNA pellet was washed with 70% ethanol and resuspended in 20 µL of sterile water. All purified DNA was quantified by spectrophotometry using a Nanovue (GE Healthcare, Little Chalfont, UK) and diluted to a 10 ng/µL concentration.

The qPCR reactions were set up using SsoFast EvaGreen Supermix (BioRad, Hercules, CA, USA) in 10 µL reactions, using 0.5 nM of each primer and 10 ng of total DNA per reaction in a Rotorgene Q (Qiagen, Hilden, Germany) using a 72 tubes rotor. The primers used target the *fluc* gene (FLUCFWR and FLUCREV, Table 1) and amplify a 150 bp target. A standard curve was done using known concentrations of the p2100P69FLUC plasmid in concentrations ranging from 10 ng to 10 fg at 1 log steps in quadriplicates. Threshold level for CT determination was 0.01889 and the efficiency of the reaction was 1.0744. Melting curves for each reaction were conducted from 60 to 98 °C in 0.5 °C steps, resulting in an amplicon melting temperature of 82.5 °C.

### 2.5. Viability and Caspase Activity Measurements

To measure insect cell viability and effector caspase activity, we utilized the ApoLive-Glo kit (Promega) following the manufacturer’s recommendations. We plated 2 × 10^4^ of UFLAg, Tn5B, Sf9, Ld652Y, Chch and Bm5 cells in 96 well opaque dark plates and infected the cells with AgMNPV-2D virus at MOI 10; after 1 h incubation for virus adsorption to the cells, the cell culture medium was replaced with 100 µL of new media and the time course of infection was then started. To account for background levels of fluorescence and luminescence we also plated and measured 100 µL of cell medium only at each time point and subtracted the background luminescence and fluorescence values from the infected cell measurements. At 0, 24, 48 and 72 hpi the viability of infected cells was estimated by adding 50 µL of the viability reagent GF-AFC substrate per well, incubated for 30 min and measured the fluorescence in a SpectraMax M2 spectrophotometer (Molecular Devices, Sunnyvale, CA, USA) followed by addition of 100 µL of the Caspase-glo^®^ 3/7 reagent, incubation for 30 min, followed by luminescence measurement in a Glomax 96 luminometer (Promega) with 1 s integration time per well.

## 3. Results

### 3.1. Permissive Cell Lines

**Figure 2:**
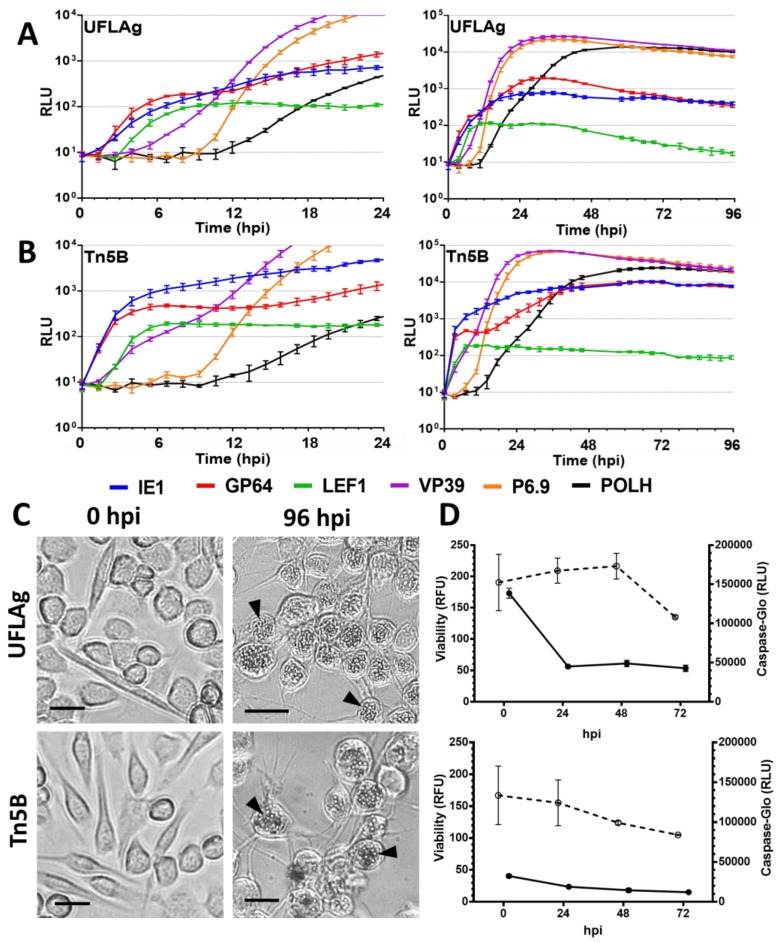
Infectivity profile of AgMNPV to UFLAg and Tn5B insect cell lines. (**A**) and (**B**) Measurement by real time luciferase assay of luciferase expression derived from different AgMNPV promoters during infection of permissive cells UFLAg (**A**) and Tn5B (**B**) at MOI 10. Insect cell growth media was supplemented with D-luciferin and the measurement was done at 15 min intervals in a non-destructive fashion. Horizontal axis in hours post infection (hpi). Error bars represent standard deviation of the mean of three independent replicates. RLU: Relative Light Units. (**C**) Light microscopy of infected cells at 0 and 96 hpi. Black arrows indicate occlusion bodies (OBs) in cell nucleus. Scale bars = 25 µm. (**D**) Cell viability and effector caspase activity of each cell line during 72 h of infection with wild-type AgMNPV at MOI 10, ○ = Viability in Relative Fluorescence Units (RFU) and ● = Caspase activity in Relative Light Units (RLU), error bars represent standard deviation to the mean of three independent replicates.

We found that in the permissive cell lines UFLAg and Tn5B, prIE1 and prGP64 have similar expression profiles during the first 3 h of infection (Figure 2A,B), characteristic of immediate early promoters. In the case of Tn5B cells, prIE1 is detected before 1 h p.i, immediately followed by prGP64 (Table 2). In contrast, prLEF1 takes at least one and a half hours more than prIE1 to express detectable amounts and present the weakest expression of all promoters, peaking at 12–18 hpi and subsequently reducing signal levels (Figure 2A,B, UFLAg and Tn5B, green lines, Table 3 and Table 4). The VP39 promoter starts expression at similar times as prLEF1, between 2 and 4 hpi, thus classifying both as delayed-early promoters (Table 2). However, after 12 hpi, the luminescence levels of prVP39 increased drastically, characteristic of late promoter hyperexpression. The P6.9 promoter presented the profile of a late promoter, with first detection after 9 hpi and steep slope indicating fast accumulation of intracellular luciferase. Both prVP39 and prP6.9 peaked at 36 hpi, inducing the highest luminescence levels of all promoters (Table 4). The very late POLH promoter was the last to activate, starting after 12 hpi and peaking at 70 hpi, with a slower rate of expression than prP6.9. At 96 hpi, abundant polyhedra are observed in the nucleus of infected UFLAg and Tn5B cells (Figure 2C).

Between 6 and 12 hpi, as the late phase begins, the late promoter elements are activated (i.e., prP6.9). The GP64 promoter presented a second phase of increased expression after 9 hpi Also, it is in this moment that the slope of the prVP39 curve aligns with the start and slope of the prP6.9 curve (Figure 2A,B). After luminescence levels reached their peaks, there is a plateau representative of a reduction in the rate of luciferase synthesis. A distinction is made that UFLAg presented 30 h earlier expression peak times for prIE1 and prGP64, compared with Tn5B (Table 3). The LEF1 promoter is the only one that is not affected positively by the beginning of the late phase; its levels drop continuously after reaching peak intensity, between 12 to 18 hpi (Figure 2A,B, Table 3).

### 3.2. Semipermissive Cell Lines

**Figure 3:**
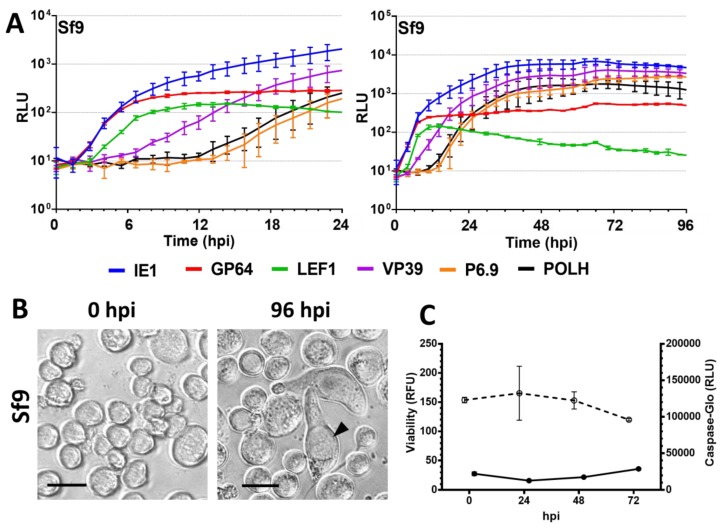
Infectivity profile of AgMNPV to Sf9 insect cell lines. (**A**) Measurement by real time luciferase assay of luciferase expression derived from different AgMNPV promoters during infection of Sf9 semipermissive cells at MOI 10. Insect cell growth media was supplemented with D-luciferin and the measurement was done at 15 min intervals in a non-destructive fashion. Horizontal axis in hours post infection (hpi). Error bars represent standard deviation of the mean of three independent replicates. RLU = Relative Light Units. (**B**) Brightfield microscopy of infected cells at 0 and 96 hpi Black arrows indicate enlarged nucleus of infected cells with no OBs. Scale bars = 25 µm. (**C**) Cell viability and effector caspase activity of each cell line during 72 h of infection with wild-type AgMNPV at MOI 10, ○ = Viability in Relative Fluorescence Units (RFU) and ● = Caspase activity in Relative Light Units (RLU), error bars represent standard deviation to the mean of three independent replicates.

The semipermissive Sf9 cell line presented similar start times of early promoters compared with permissive UFLAg (Figure 3A). Distinctions from the permissive cell lines occur in the prGP64 expression curve that peaked at 10 times lower the level of prIE1 peak expression. The late promoters prVP39, prP6.9 and prPOLH failed to peak above the IE1 promoter peak expression level, indicating that there was no hyperexpression of these elements. These also presented onset times at 2 to 4 h later and peak times at 20 h or more later than in permissive cells (Figure S1). Large cells with enlarged nucleus were the predominant morphotype at 96 hpi but no OBs were observed (Figure 3B).

### 3.3. Nonpermissive Cell Lines

**Figure 4:**
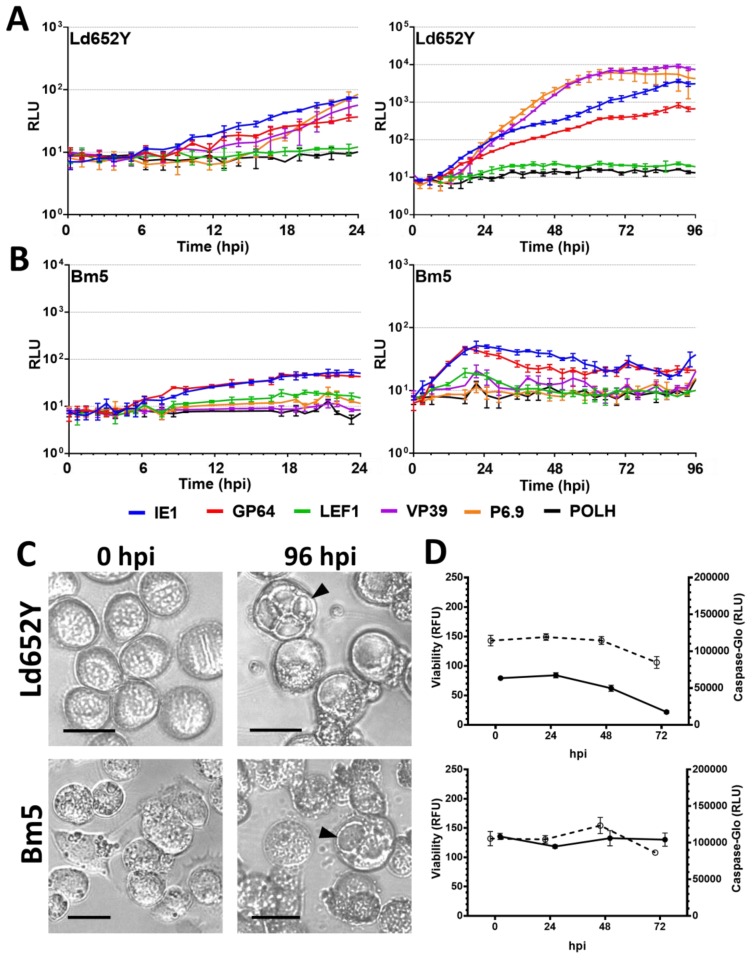
Infectivity profile of AgMNPV to Ld652Y and Bm5 insect cell lines. (**A**) and (**B**) Measurement by real time luciferase assay of luciferase expression derived from different AgMNPV promoters during infection of nonpermissive cells Ld652Y (**A**) and Bm5 (**B**) at MOI 10. Insect cell growth media was supplemented with D-luciferin and the measurement was done at 15 min intervals in a non-destructive fashion. Horizontal axis in hours post infection (hpi). Error bars represent standard deviation of the mean of three independent replicates. RLU = Relative Light Units. (**C**) Brightfield microscopy of infected cells at 0 and 96 hpi Black arrows indicate vacuoles in infected cells. Scale bars = 25 µm. (**D**) Cell viability and effector caspase activity of each cell line during 72 h of infection with wild-type AgMNPV at MOI 10, ○ = Viability in Relative Fluorescence Units (RFU) and ● = Caspase activity in Relative Light Units (RLU), error bars represent standard deviation to the mean of three independent replicates.

The nonpermissive Ld652Y cell line presented an overall delayed promoter expression profile (Figure 4A). The IE1 promoter was only activated at 12 hpi, followed by prGP64 at 13 hpi and prVP39 and prP6.9 at 17 hpi This represents a delay of 10 h over the activation of the early promoters on permissive cell lines (Figure S1). Moreover, expression based on prLEF1 and prPOLH does not rise above the baseline of 20 RLU. The VP39 and P6.9 late promoters expression occurred at a slower rate, but were able to induce expression levels of luciferase that are equivalent to the same promoters in the permissive cell lines at 10^4^ RLU levels. Light microscopy evaluation revealed that cell rounding and nucleus enlargement occurs during the first 48 hpi, but by 96 hpi no OBs were observed and a large proportion of cells presented large vacuoles (Figure 4C).

The nonpermissive Bm5 cell line weakly activated the early promoters (prIE1 and prGP64) within the first 24 hpi (Table 2), but no other promoter was activated during the 96 h of infection (Figure 4B). Some cytopathic effects, such as cell rounding and nucleus enlargement were observed within the first 24 hpi but it subsided; while at 96 hpi, cell membranes with a rugged appearance and many cells with large vacuoles are observed (Figure 4C). No polyhedra formation was detected at 96 hpi

**Figure 5:**
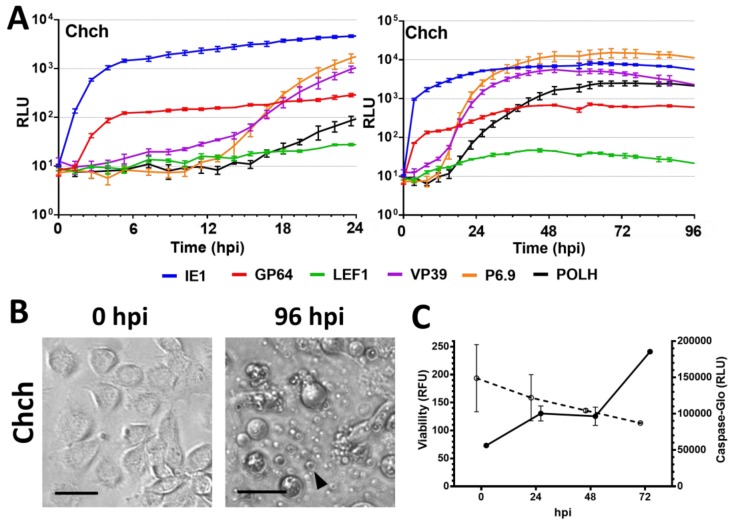
Infectivity profile of AgMNPV to Chch insect cell lines. (**A**) Measurement by real time luciferase assay of luciferase expression derived from different AgMNPV promoters during infection of nonpermissive Chch cells at MOI 10. Insect cell growth media was supplemented with D-luciferin and the measurement was done at 15 min intervals in a non-destructive fashion. Horizontal axis in hours post infection (hpi). Error bars represent standard deviation of the mean of three independent replicates. RLU = Relative Light Units. (**B**) Brightfield microscopy of infected cells at 0 and 96 hpi Black arrow indicates apoptotic bodies. Scale bars = 25 µm. (**C**) Cell viability and effector caspase activity of each cell line during 72 h of infection with wild-type AgMNPV at MOI 10, ○ = Viability in Relative Fluorescence Units (RFU) and ● = Caspase activity in Relative Light Units (RLU), error bars represent standard deviation of the mean of three independent replicates.

The nonpermissive Chch cell line undergoes apoptosis after AgMNPV infection starting at 12 hpi, becoming prevalent overtime in most cells at 96 hpi, as observed by light microscopy (Figure 5B). This cell line presented quick activation of early promoters (prIE1 and prGP64), but the delayed early promoter prLEF1 only activated at 18 hpi (Table 2) and maintained luminescence levels close to the baseline throughout the assay (Table 4). Late promoters (prVP39 and prP6.9) presented delayed activation times (Table 2) and never reached levels above prIE1, similar to the profile of Sf9 (Figure S1), while levels of prPOLH peaked at 10 times lower levels than prP6.9 (Figure 5A). No polyhedra were observed at 96 hpi

### 3.4. Viral DNA Quantification by qPCR

**Figure 6:**
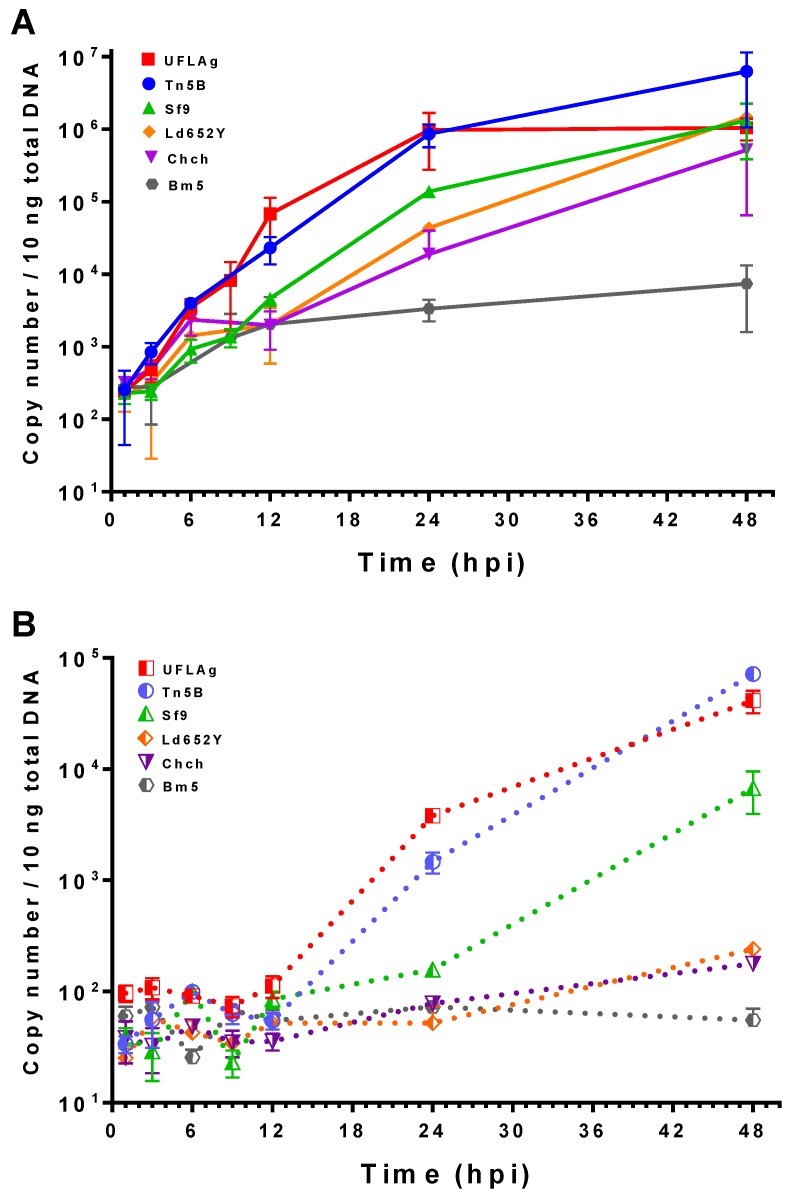
Quantification of intracellular viral DNA replication (**A**) and extracellular budded virus (BV) DNA (**B**) in Tn5B, UFLAg, Sf9, Ld652Y, Chch and Bm5 insect cells during AgMNPV infection. Error bars represent standard deviation of the mean of three independent replicates.

To complement gene expression data, we quantified virus replication and cell-free extracellular viral DNA in the form of matured extracellular virus (BV) by absolute qPCR. By 6 hpi, the concentration of intracellular viral DNA had no significant difference amongst all cell lines and time points (*t* test, *p* > 0.01, Figure 6A). After 6 hpi, viral DNA replication in the permissive Tn5B and UFLAg cell lines increased above baseline levels of previous time points to peak near 10^6^ copy number levels at 24 hpi Extracellular viral DNA was detected only after 12 hpi, reaching levels of 10^4^ to 10^5^ copy numbers at 48 hpi (Figure 6B).

Viral DNA replication in the semipermissive Sf9 cell line was detected first between 12 and 24 hpi, reaching levels 10-fold lower than permissive cell lines at 24 hpi At 48 hpi intracellular viral DNA is not significantly different than UFLAg and Tn5B (*t* test, *p* > 0.01). The levels of BV DNA at 24 hpi remained similar to baseline levels (*t* test, *p* > 0.01), rising only after 24 hpi to levels at least 10-fold lower than permissive cells. The interval between viral DNA replication and viral extracellular DNA first detection times was smaller in Tn5B and UFLAg cells (from 9 to 24 hpi) when compared to Sf9 cells (24 to 48 hpi).

The nonpermissive Chch and Ld652Y cell lines also presented a delayed replication starting from 12 hpi that reached significant levels at 48 hpi; however, no significant amount of extracellular BV DNA was detected at the same time point. The nonpermissive Bm5 cell line did not amplify intracellular viral DNA or form sufficient extracellular BV DNA for detection within 48 hpi

### 3.5. Viability and Caspase Activity Assay

We observed that there is a significant decrease in the viability of the cell lines UFLAg (Figure 2D), Tn5B (Figure 2D), Sf9 (Figure 3C), Ld652Y (Figure 4D) and Chch (Figure 5C) as infection progressed (*t* tests between 0 and 72 hpi, *p* < 0.001), but no significant decrease in viability was detected for the infection of the Bm5 cell line (Figure 4D, *t* test, *p* > 0.01).

Caspase activity decreased overtime during AgMNPV infection of UFLAg (Figure 2D), Tn5B (Figure 2D) and Ld652Y (Figure 4D), which is consistent with the inhibitory action of the anti-apoptotic *iap3* genes over the effector caspase pathway. The Sf9 cell line presented a small increase of caspase activity overtime (Figure 3C), but levels were considerably low. The Chch cell line presented a dramatic increase in caspase activity from 48 to 72 hpi (Figure 5C), consistent with microscopy observations of the apoptosis phenotype and confirming that the AgMNPV anti-apoptotic protein is not capable of inhibiting the effector caspase pathways of Chch. The Bm5 cell line presented high and stable caspase activity overtime that remained stable (Figure 4D).

## 4. Discussion

### 4.1. Methods for the Investigation of Baculovirus Promoter Activity

Initial characterization of isolated baculovirus promoters used the chloramphenicol acetyl transferase (CAT) enzyme as a reporter gene [47,48,49,50,51]; however, this technique utilizes radioactive compounds to quantify reporter gene expression. Another alternative to measure promoter activity is the use of fluorogenic proteins, such as the green fluorescent protein (GFP). This analysis depends on the excitation of the proteins by light beams and suffers from excessive backgrounds from various fluorogenic compounds present in insect cell media and insect cells [52,53,54]. Luciferase-based reporter assays have as a benefit a low background in insect cells.

The standard method of promoter evaluation using luminescence measurement involves lysing the cells after treatment, followed by addition of the chemilluminescent reaction substrate. In the real time luminescence measurement assay described in this work, the addition of D-luciferin to the growth medium allowed for continuous measurement of the same population of cells over time. The different cells maintained in different culture media with D-luciferin supplementation showed no observable toxic effects to uninfected cells, as determined by light microscopy evaluation. For proper comparison between treatments, we standardized the cell number and used a high MOI (10) to infect all cells synchronously. The non-lytic nature of the experiment simplified automated data collection yielding a higher number of collection points in time, which allowed for a more dynamic and detailed picture of the progression of virus infection to emerge.

Another way to analyse gene expression is by transcriptomic analysis. However, the amount of mRNA produced does not correlate directly with the amount of protein that is translated [55]. Chen et al. [26] pointed out that due to the small intergenic regions between baculovirus genes and also the overlapping nature of viral mRNAs, the global analysis of transcription during baculovirus infection is very difficult. While transcriptomic analysis describes the multitude of transcribed mRNA derived from genomic expression in a cross-sectional approach, isolated promoter analysis using a reporter gene has a greater potential to unravel how these mRNAs are converted into proteins. Hopefully, this longitudinal and sensitive approach to data collection will complement the baculovirus literature by revealing more subtle expression patterns.

### 4.2. Temporal Expression Patterns of Individual Promoters and Correlations with Viral Replication in Permissive Cell Lines

Baculovirus cell infection is characterized by a succession of events determined by gene expression that are transcriptionally controlled. It begins with the expression of the immediate early 1 gene (*ie1*), which is an essential gene transcribed by host RNA pol II within the first hour of infection by AcMNPV in Sf21 insect cells [56]. The resulting protein acts as a transactivator of viral gene expression [17] and is one of the primers for the formation of the virogenic stroma within the nucleus [57,58]. Our results show that the first promoter to be active in permissive, semi and nonpermissive cell lines was prIE1, exceptionally in Tn5B detected at 27 min p.i., which as far as we know, is the fastest known in vitro detection of protein expression driven by this promoter in the literature. The ability to express genes at a high pace and intensity in vivo is advantageous in the field since it allows the virus to avoid host defenses and guarantees abundant BV production which helps to spread the infection in the host larvae.

The expression of the major envelope glycoprotein GP64 at immediate early times has been implicated in an interesting phenomenon during primary infection of larval midgut cells, where multiple enveloped ODVs would be able to cross through the basal laminae of the midgut faster than the onset of viral replication [59]. This phenomenon is attested by the detection of the protein at early times post infection [60,61] and the increased oral lethality and speed of spread on larval tissues when the GP64 ORF is under the control of an early promoter [62]. The onset and the first hours of AgMNPV prIE1 and prGP64 expression curves are very similar and this places both within the immediate early promoter class in terms of timing. In permissive cells, prGP64 presented a clear bimodal pattern of expression which is very similar to the description of AcMNPV’s GP64 bimodal gene expression [62,63]. It is very likely that the subsequent burst of prGP64 of the AgMNPV is a result of the activation of the −170 bp late TSS motifs after viral DNA replication, as described for AcMNPV [50,63] and OpMNPV [60], since this motif is also conserved in the AgMNPV. Another peculiarity of this promoter is that the late burst of expression is not as intense as other late promoters (i.e., prVP39 and prP6.9).

In permissive cell lines, prLEF1 presented a profile of a promoter at delayed early times with weak expression. This promoter controls the primase component of the viral DNA polymerase complex [20,31], which is essential for viral replication, and due to its function is a protein required at the early phase. The low expression generated by prLEF1 in all cell lines suggests that the LEF1 protein is required only at low amounts within the cell to induce viral replication. This promoter represents a class of transcriptional controllers that are probably tuned to express accessory proteins needed only in small amounts during infection. It lacks a TATA box near the 2 CAGT TSS motifs at −53 and −79 bp, which may explain the low expression efficiency at early times. This is consistent with AcMNPV *lef1* gene expression, that is detected at a delayed early moment, beginning at 3 hpi [64] and peaking at low levels at 12 hpi with reduced transcription [26] or no detectable transcripts after 24 hpi [64]. It is puzzling to observe the presence of a TAAG late motif at −171 bp that appears to be inactive, in contrast with prGP64 AgMNPV prLEF1. Transcriptomic data of AcMNPV infection suggests that most of these late TSS are utilized at late times post infection [26]. The nucleotide context surrounding this late motif within prLEF1 may be responsible for the suppression of this TSS.

The major late TSS motif found in most AcMNPV late gene promoters is the nucleotide sequence TAAG, present in promoters of capsid related structural genes (e.g., *vp39*, *p6.9*) [65,66,67], and very late genes, such as *polh* [68,69]. This late motif is present within 50 bp upstream of ORF start codon of AgMNPV`s *vp39*, *p6.9* and *polh* promoters. All late promoters are expected to be active only after viral DNA replication begins and this work demonstrates this connection, easily observed in permissive cell lines that initiate viral replication after 6 hpi and activate the late promoters subsequently (i.e., prP6.9, prPOLH). Hyperexpression of the late promoters was consistently observed as higher levels of peak expression in comparison with the early promoters, and this correlated with high concentrations of extracellular virus produced by these cells.

The presence of the VP39 protein within the nucleus at delayed early moments contributes to the formation of the virogenic stromae [57] and at late times the high expression levels are essential to guarantee abundant virion formation. The capsid gene promoter VP39 is a bimodal promoter with a weak delayed early and strong late expression profile. This mirrors the profile of prVP39 driven expression observed in permissive cells infected by AgMNPV. This promoter lacks an early CAGT TSS motif but possess 2 TATA box motif at −41 and −89 bp from ORF start codon. The TATA box motif is sufficient for early promoter transcription during AcMNPV infection [27,50] but there is no evidence of the actual early transcription start site for the AgMNPV prVP39. The AcMNPV *vp39* gene was described as a late gene due to the dependency of viral DNA replication [65] and time of detection with maximal transcription between 12 to 24 hpi [26,49,70], but it has also been described as having a delayed early component [67]. We can confirm the high levels of expression of AgMNPV`s prVP39 in permissive cells at late times but the proper classification for this promoter would be a bimodal delayed early and late promoter.

The P6.9 gene promoter controls the expression of the basic P6.9 viral DNA binding protein that helps virion formation by condensing replicated viral DNA prior to encapsidation [66] and is one of the most abundantly expressed genes during AcMNPV infection of the Tn5B cell line [26,71]. The AgMNPV prP6.9 promoter was profiled as a late promoter with the highest expression levels amongst the isolated promoters in this work. The AgMNPV prP6.9 possess only a single late TAAG motif at −30 bp and two distant TATA box motifs (−143 and −273 bp). In contrast with prVP39, the distant TATA box motifs in prP6.9 were not sufficient for early promoter activation. Another conclusion that can be made is that there is no accumulative contribution of multiple (prVP39) in comparison with single (prP6.9) late TSS motifs to total expression, since the curves of these two promoters were very similar at late times and reach similar luminescence values.

The *polh* gene is abundantly transcribed at very late infection times [26] and the resulting polyhedrin protein is the main component of the crystal lattice that forms the occlusion bodies [72]. It is controlled by the very late *polh* gene promoter, that, in AgMNPV, possesses a single late TAAG motif at −44 bp and a TATA box element at −82 bp. The similarity of known motifs with other late promoters (i.e., prVP39, prP6.9) would imply a similar onset of expression at 9 hpi However, during AgMNPV infection, prPOLH consistently has its time of activation delayed through an unknown mechanism. It is important for the baculovirus infection to separate the late and very late phases in time to permit virion migration from the nucleus to the cell membrane and thus form the BV phenotype, which would promote the spread of infection to other cells. This would be followed by the expression of very late genes that induces virion retention in the nucleus to be occluded into the crystal matrix of the polyhedra phenotype.

Of all promoters evaluated during permissive host cells infection, prP6.9 and prVP39 were shown to be the most productive in terms of driving heterologous gene expression. During AgMNPV infection in permissive cell lines, the late promoters peak near 36 hpi on at least 1 log order of magnitude above early promoters and also peak 24 h earlier than prPOLH. This represents significant gains in time and resources, as well as a higher quality of the recombinant protein yield. Transcriptomic studies have also shown evidence that these two genes are the most abundantly expressed during AcMNPV infection [26,73] and there may be distinct benefits of *vp39* and *p6.9* gene promoters over *polh* gene promoters in protein expression vectors [51,54,67,71].

### 4.3. The Semipermissive Cell Line Sf9 and Restriction of Very Late Promoter Expression

The semipermissive Sf9 cell line presented similar start times for all early promoters compared with permissive cell lines. The late promoters prVP39, prP6.9 and prPOLH presented a significant delay in onset and peak times, failed to peak at significantly higher levels of the early IE1 promoter expression curve and there was no late burst of expression of the bimodal prGP64. Quantification of AgMNPV gene transcription by RT-qPCR also reported that late gene expression in UFLAg cells had an overall faster onset and higher transcription levels than in Sf9 [74]. The delays observed for the prP6.9 promoter on the time of onset correlate well with a delay in intracellular replication. The lower expression levels of late promoters prVP39 and prP6.9 also correlate with lower concentrations of extracellular virus, suggesting that in semipermissive cell lines these promoters fail to induce sufficient synthesis of structural proteins to generate high titers of BVs. In a similar fashion, the low expression levels of prPOLH suggests that Sf9 fails to form OBs due to reduced expression from this promoter; however, the exact mechanisms responsible for limited late and very-late promoter expression are not well known. The hyperexpression of viral genes in permissive—or lack thereof in semipermissive—cells may be the result of a specific interaction between host and viral regulatory proteins of the late phase, such as the *fp25k* gene of AcMNPV, known to modulate *polh* expression and the formation of matured OBs differentially in varied cell lines and host tissues [75,76].

### 4.4. Viral Infection in Nonpermissive Cell Lines and the Effects on the Promoters

The nonpermissive Ld652Y cell line presented a profile with systemic delays on the onset of the expression from all promoters and a slow rise of luminescence levels compared with permissive cell lines; this also correlates with the delayed onset of replication of viral DNA. The late promoters prVP39 and prP6.9 were activated and induced high expression levels; however, no extracellular virus was detected at that time point. Moreover, the prLEF1 promoter barely rises above baseline and the POLH promoter appears not to be activated at all. This delayed pattern of expression in Ld652Y may be related to the global protein synthesis shutdown defense mechanism described for AcMNPV infection [77,78]. This phenomenon was characterized as reduced viral and host gene expression at late times that results in reduced matured virion formation and a reduction in cell viability [79]. This cellular response may be countered by a viral factor, the *hrf-1* gene of *Lymantria dispar* MNPV, that rescues the ability of AcMNPV to fully infect Ld652Y cell line [78,80]. It has also been shown that the viral inhibitor of apoptosis p35 of AcMNPV indirectly activates the global translation arrest by inhibiting the caspase effector pathway [81]. The AgMNPV genome lacks *p35* but utilizes the *iap3* gene to block cell apoptosis [8,19]. The observation that caspase activity decreases during AgMNPV infection suggests that *iap3* is functional against Ld652Y effector caspase homologues. By blocking this pathway, AgMNPV infection may also lead to the activation of the protein synthesis shutdown in Ld652Y. The only distinction to previous observations of this phenomenon is that what was described for AcMNPV infections was a global *shutdown* of gene expression in infected cells beginning at the early phase [81]. The results we obtained presented a slow and continued increase of protein expression, suggesting that a proper description would be a *limitation* in expression rather than a complete blockage of protein synthesis.

This work presents the first description of AgMNPV infection in the recently established Chch cell line. This cell line is not permissive to AcMNPV infection, undergoing apoptosis after infection [37]. We have observed that the AgMNPV baculovirus is not capable of inhibiting the caspase pathway and it induces apoptosis in Chch. Amazingly, this cell line presents a similar promoter expression profile to semipermissive Sf9 cell line during infection, with strong immediate early gene expression but delayed and limited late and very-late promoter expression. Although apoptosis is not capable of blocking viral DNA replication or the transcription from late promoters, it does disrupt the formation of both BV and OBs possibly at the level of virus assembly within the nucleus. Since the group II Alphabaculovirus *Trichoplusia ni* SNPV and *Chrysodeixes chalcites* SNPV do not induce apoptosis in this cell line [37], while two distinct group I Alphabaculoviruses do, it is suggestive that the antiapoptotic genes have a narrow range of targets and may be strong determinants of the host range of a virus.

The nonpermissive Bm5 cell line presented a unique expression profile, activating only the immediate early prIE1 and prGP64 that peaks at a very low level within 24 hpi, without any activity from the other promoters. Delayed early promoter activity is not significant and this implies that IE1 transactivation is compromised. Lack of viral DNA replication and expression from late and very late promoters further indicates that the infection of AgMNPV does not progress beyond the delayed early phase. All of this is consistent with previous observations that AgMNPV infection in this cell line is completely abortive [13,14]. This profile is also similar to what was described for AcMNPV`s infection in Bm5 [82]. In this case the ability of the virus to replicate was rescued when a fragment of the *helicase* gene of *Bombyx mori* NPV (BmNPV) replaced its homologue in the AcMNPV genome [83]. All of these observations point to the presence of an effective cellular defense against baculovirus infection and/or incompatibility between viral factors and the host environment in Bm5 that results in the inability of the AgMNPV to replicate its DNA and progress through the late phase of the infection. This is distinct from apoptosis and the global translation arrest observed in Chch and Ld652Y, respectively. With further work with the AgMNPV baculovirus, we will be able to assess if its ability to properly infect this cell line may also be rescued by the *helicase* gene of BmNPV.

The limited expression of the prPOLH element in semipermissive and nonpermissive cell lines correlates with a lack of OB formation and this may function as a selective defense mechanism of a nonpermissive host in a broader sense, since it would result in lower concentrations of infective polyhedra in the field and thus reduce viral spread considerably.

## 5. Conclusions

In this work, we characterized selected AgMNPV promoters of each phase of the cellular infection in cell lines with different susceptibilities to infection using a new methodology that is the real time detection of luminescence in living cells. It is a powerful and simple technique that presents a dynamic and detailed picture of promoter specific gene expression during cell infection. Using this technique, we were able to properly distinguish between the onset of expression of immediate early and delayed early promoters and the onset of late and very late promoters. The sequence of promoter activation in permissive cell lines during AgMNPV infection was prIE1, prGP64, prLEF1, prVP39, prP6.9 and prPOLH. This sequence and the onset of expression are consistent with the functions of the proteins they control. The activation of late promoters correlated positively with viral DNA replication within the cells, while the hyperexpression derived from these promoters in permissive cell lines correlates positively with higher concentrations of extracellular viruses. The sequence of promoter activation was conserved between permissive, semipermissive and nonpermissive cell lines. However, nonpermissive cell lines presented disrupted expression profiles with delays in the activation of promoters and reduced late and very-late promoter expression. This technique has great potential and may help to advance the understanding of the regulation of baculovirus gene expression, the cellular defenses against an infection and the transcriptional mechanisms associated with the *cis* elements contained within the promoters. It will also be useful for the optimization of protein expression using the baculovirus expression vector systems that are available.

## Figures and Tables

**Table 1:** List of primers used in this work. In italics the added restriction enzyme sequences (AGCTT for *Hind*III or CCCGGG for *Xma*I). Underlined is the PolyA tail signal sequence added to the 3′ of the *luciferase* gene. AgMNPV genbank accession number: NC_008520.

Target	Primers	Primer Sequence	AgMNPV Genome Coordinates	Amplicon Size (bp)
prIE1	FWRHind	CCCC*AAGCTT*GAATTGTCGGTGAGCGTTGCGCGT	121,218−121,652	466
	REV	GCTATGCACGCGCAATCCG		
prGP64	FWR	GGATCCATTTTGATGAAGGTCTT	106,097–106,446	360
	REV	AGATCTTTGTTATGTCTTGTAGC		
prLEF1	FWR	GTTGCGGCTTGACCACGG	14,848–15,104	260
	REV	GGATCCTTGTAGGGCGTCTA		
prVP39	FWRHind	AAGC*AAGCTT*TTTCGCGCCACACAAGCGGCACCAACG	71,414–71,703	290
	REVXma	TTA*CCCGGG*TTTGCTACAATGGACGACTTTGTGATT		
prP6.9	FWRHind	ACTG*AAGCTT*TCGCCAGCCCTGTGATGCGTTACG	81,693–82,175	483
	REVXma	ATTA*CCCGGG*AAGTGTTTTACAATGTAGCTTTAA		
prPOLH	FWRHind	AAGC*AAGCTT*ATTTGGAGTGTTTGTACGATT	729–1149	421
	REVXma	ATTA*CCCGGG*AGTTATAGCAAATTTTACTACAAAG		
*fluc*	qPCRFWR	AAACGCTGGGCGTTAATCAG	-	145
	qPCRREV	TCGTCCCAGTAAGCTATGTC		
*fluc*	FLUCFWR	GATTTAGGTGACACTATAG	-	1807
	FLUCREV	AAGGGATCCAGCTCGATTTATTCGACCTCGA		

**Table 2:** Time points (in h: min p.i.) of first detection derived from recombinant AgMNPV infection of insect cells. Time values are averages and standard deviations (Avg and SD) of three independent biological replicates. First detection is defined as first detected luminescence above baseline level of 20 RLU.

Cell Line	Virus											
	IE1FLUC		GP64FLUC		LEF1FLUC		VP39FLUC		P6.9FLUC		POLHFLUC	
	Avg	SD	Avg	SD	Avg	SD	Avg	SD	Avg	SD	Avg	SD
**UFLAg**	1:58	0:13	2:03	0:18	3:42	0:05	5:35	0:39	10:10	0:08	12:49	0:13
**Tn5B**	0:27	0:05	0:32	0:02	2:15	0:10	2:03	0:12	9:08	0:28	13:17	0:48
**Sf9**	2:03	0:13	2:03	0:04	3:51	0:03	6:39	0:11	15:08	1:36	16:53	0:55
**Ld652Y**	11:54	0:34	13:19	0:16	-	-	17:07	2:22	17:25	1:06	-	-
**Bm5**	8:30	0:04	6:46	0:36	-	-	-	-	-	-	-	-
**Chch**	1:46	0:12	1:55	0:08	18:09	1:38	8:52	0:18	13:47	0:33	17:38	0:56

**Table 3:** Time points (in h: min p.i.) of peak values derived from recombinant AgMNPV infection of insect cells. Time values are averages and standard deviations (Avg and SD) of three independent biological replicates.

Cell Line	Virus											
	IE1FLUC		GP64FLUC		LEF1FLUC		VP39FLUC		P6.9FLUC		POLHFLUC	
	Avg	SD	Avg	SD	Avg	SD	Avg	SD	Avg	SD	Avg	SD
**UFLAg**	32:15	3:18	31:58	3:21	18:41	10:30	36:58	1:55	34:54	1:24	59:24	0:26
**Tn5B**	66:25	0:14	65:19	2:29	12:31	3:01	36:05	1:09	38:26	1:32	72:10	0:55
**Sf9**	63:09	1:56	76:49	8:52	12:45	0:50	76:01	8:19	89:47	4:01	88:59	1:16
**Ld652Y**	109:18	2:24	110:59	0:16	-	-	90:30	0:41	74:45	11:20	-	-
**Bm5**	57:33	30:20	17:57	1:14	-	-	-	-	-	-	-	-
**Chch**	63:22	1:40	52:39	6:51	46:13	3:57	49:57	0:54	70:01	2:59	77:27	10:59

**Table 4:** Peak luminescence values (in relative light units, RLU) derived from recombinant AgMNPV infection of insect cells. RLU values are averages and standard deviations (Avg and SD) of three independent biological replicates.

Cell Line	Virus											
	IE1FLUC		GP64FLUC		LEF1FLUC		VP39FLUC		P6.9FLUC		POLHFLUC	
	Avg	SD	Avg	SD	Avg	SD	Avg	SD	Avg	SD	Avg	SD
**UFLAg**	804	94	1998	91	130	5	27170	1512	22339	2278	13997	601
**Tn5B**	10768	533	10209	780	204	16	72005	1483	68130	2111	24660	1617
**Sf9**	7087	1187	583	19	158	11	4210	1250	2875	255	387	61
**Ld652Y**	4652	625	1210	137	18	1	9562	883	6483	2329	17	0
**Bm5**	69	28	50	3	22	2	19	1	21	3	18	1
**Chch**	8257	726	739	25	52	1	5651	786	15364	4115	2586	354

## Supplementary Materials

The following are available online at www.mdpi.com/1999-4915/9/6/132/s1, Figure S1: Expression profiles of individual promoters in different cell lines.
