# Supplementary material for: Real-Time Expression Analysis of Selected Anticarsia gemmatalis multiple nucleopolyhedrovirus Gene Promoters during Infection of Permissive, Semipermissive and Nonpermissive Cell Lines
Source: Viruses. 2017 May 31;9(6):132. doi: 10.3390/v9060132 (PMC5490809; doi:10.3390/v9060132)
Supplement: Supplementary file 1 [file viruses-09-00132-s001.docx]

**Figure S1.** Expression profiles of individual promoters in different cell lines. Insect cell growth media was supplemented with D-luciferin and each cell line was infected with a recombinant AgMNPV at MOI 10. Lines represent the average of three replicates at 30 min intervals. Horizontal axis in hours post infection (hpi). RLU = Relative Light Units.
